# State-Level Variation in and Barriers to Medicaid Abortion Coverage

**DOI:** 10.1001/jamanetworkopen.2025.30804

**Published:** 2025-09-08

**Authors:** Jasmine W. Jiang, Sarah J. Ho, Sakinah C. Suttiratana, Carlisle E. W. Topping, Arjun K. Venkatesh, Rieham Owda, Priscilla J. Smith, Dalia Owda, Hazar Khidir

**Affiliations:** 1 Yale School of Medicine, New Haven, Connecticut; 2 General Internal Medicine, Yale School of Medicine, New Haven, Connecticut; 3 Department of Emergency Medicine, Yale School of Medicine, New Haven, Connecticut; 4 Center for Outcomes Research and Evaluation, Yale School of Medicine, New Haven, Connecticut; 5 Department of Obstetrics and Gynecology, University of Michigan Medical School, Ann Arbor; 6 Yale Law School, New Haven, Connecticut; 7 Department of Emergency Medicine, Henry Ford Health System, Detroit, Michigan

## Abstract

**Importance:**

Approximately 35% of individuals seeking abortion care use Medicaid for health insurance. Although the Hyde Amendment restricts use of federal funds for most abortions, states can supplement coverage using state funds. Understanding the scope of abortion coverage across states and potential barriers to access may help address health care inequities and inform interventions.

**Objective:**

To characterize state Medicaid abortion policies by conducting a qualitative analysis of publicly available state documents on Medicaid policy.

**Design, Setting, and Participants:**

This qualitative study analyzed Medicaid abortion policies across all 50 states and the District of Columbia (hereinafter, states). Data were systematically collected from publicly available Medicaid documents and state websites from May 2023 to February 2024.

**Main Outcomes and Measures:**

The main outcomes were key themes and descriptive statistics reporting on the scope of Medicaid abortion coverage and requirements for coverage across states, including documentation and procedures required of patients and physicians. Thematic analysis was performed to extract key themes found in abortion coverage policies, and descriptive statistics were used to show prevalence of identified themes across states.

**Results:**

The analysis of 94 documents revealed 3 key themes. First, the scope of coverage across states was heterogeneous. Eighteen states aligned with the current wording of the Hyde Amendment, 10 states described life endangerment without use of current Hyde Amendment wording, 17 states outlined additional coverage for other specified conditions for abortions, 6 states covered all abortions, and 1 state’s policy did not mention required federal coverage for rape or incest exceptions. Second, states imposed various patient restrictions and requirements with regard to abortion care coverage, with 22 states mandating reporting requirements for abortions due to rape or incest, along with other administrative hurdles for patients seeking care. Third, physicians were tasked with many responsibilities, such as determining eligibility for Medicaid abortion coverage and complying with documentation and administrative requirements. Thirty-eight states explicitly required physician certification and justification for clinical conditions warranting coverage.

**Conclusions and Relevance:**

The findings of this qualitative study of state Medicaid abortion policies suggest that there is substantial heterogeneity among states regarding the scope of Medicaid abortion coverage and that there are numerous obstacles for patients and physicians in accessing this coverage. This heterogeneity and burden may impose an additional layer of complexity to abortion access. Measures and policies that improve transparency, clarity, and efficiency may enhance access to essential abortion care for vulnerable populations.

## Key Points

**Question:**

How do different states cover abortion services under Medicaid as outlined in their respective state Medicaid policies?

**Findings:**

This qualitative study of Medicaid abortion policies using 94 documents from all 50 US states and the District of Columbia revealed substantial differences in coverage among states. The findings also highlight stringent eligibility requirements, burdens placed on patients, and numerous physician responsibilities.

**Meaning:**

The findings suggest that the heterogeneity and burden placed on patients and physicians may contribute an additional layer of complexity to abortion access for vulnerable populations.

## Teaser

This qualitative study of Medicaid abortion policies across all 50 US states and the District of Columbia analyzes abortion services and barriers to abortion coverage.

## Introduction

Abortion care is an essential component of reproductive health care, with over 900 000 abortions performed annually within the US, yet access to care still remains restricted in various states.^1^ In 2022, the Supreme Court decision in *Dobbs v Jackson Women’s Health Organization* (hereinafter *Dobbs*)^2^ overturned *Roe v Wade*^3^ and returned the legislation of abortion care to individual states. As of July 2025, 12 states have enacted total abortion bans, and 28 states have enacted abortion bans based on gestational duration.^4^

For patients, payment for abortion services has long been a barrier to abortion care, even preceding the *Dobbs* decision. The Hyde Amendment, a federal statutory provision first adopted in 1976 and renewed annually, restricts access to abortions for those enrolled in Medicaid. When first introduced, the Hyde Amendment prohibited using federal funds for any abortion “except where the life of the mother would be endangered if the fetus were carried to term.”^5^ Since then, the Hyde Amendment has evolved numerous times, with the current version listing coverage for 2 conditions: “(1) if the pregnancy is the result of an act of rape or incest; and/or (2) in the case where a woman suffers from a physical disorder, physical injury, or physical illness, including a life-endangering physical condition caused by or arising from the pregnancy itself, that would, as certified by a physician, place the woman in danger of death unless an abortion is performed.”^6^ Additionally, in 1993 and again in 1998, the Department of Health and Human Services (HHS) provided clarification to state Medicaid directors regarding the interpretation of the Hyde Amendment, writing, “All abortions covered by the Hyde Amendment, including those abortions related to rape or incest, are medically necessary services and are required to be provided by states participating in the Medicaid program.”^7,8^

While states are required to cover abortions in the scenarios outlined in the Hyde Amendment, each state has the discretion to determine the extent of abortion coverage, if any, that it is willing to provide beyond the Hyde Amendment through use of state funds. Understanding how state Medicaid policies on abortion care vary is essential, as these policies substantially impact access to abortion services and health outcomes; states that fund medically necessary abortions experience lower rates of maternal morbidity compared with those that do not.^9^ Conversely, states with more restrictive abortion policies are associated with higher maternal mortality rates.^10^ Additionally, state policies that supplement Medicaid abortion coverage beyond Hyde Amendment requirements are associated with multiple benefits for patients, including reduced patient out-of-pocket expenses, shorter appointment wait times, and decreased travel time to abortion clinics.^11,12,13^

Previous studies have examined state-level Medicaid funding for abortion care and reported which states provide Medicaid funding for abortion care, specific requirements for coverage in each state, varying degrees of restrictiveness in state policies, and inconsistencies in reimbursement rates across states, with the majority using national registry data and primary data obtained from abortion care physicians and Medicaid agency personnel.^10,14,15,16^ To our knowledge, no study to date has comprehensively analyzed the specific language and requirements in state Medicaid abortion policy documents. Such an analysis is important for understanding coverage across states and identifying potential barriers to access. Although Medicaid abortion coverage policies are limited in their application in states with abortion bans, a comprehensive analysis is important for understanding implementation of Medicaid abortion coverage across states and how policies might pose further barriers to access. Our qualitative study aimed to address this gap by examining Medicaid policies in all 50 US states and the District of Columbia with the goal of characterizing and extracting key themes from the scope of abortion coverage and coverage requirements across different states.

## Methods

### Study Design

We conducted a qualitative study to examine state-specific Medicaid abortion coverage across all 50 states and the District of Columbia (hereinafter states). The Yale University Institutional Review Board deemed the study exempt from further review, and because the study did not involve human participants, there was no need for informed consent. The study followed the Standards for Reporting Qualitative Research (SRQR) reporting guideline.

We systematically collected data from publicly available Medicaid documents and state websites between May 2023 and February 2024 (eFigure in Supplement 1). Our search strategy involved 2 tiers: a primary search and a secondary search. In the primary search, we identified state Medicaid policy manuals for providers using the keywords *manual*, *policies*, *handbook*, and *guidelines* on official state Medicaid websites. Within identified documents, we searched for the terms *abortion*, *pregnancy termination*, and *termination of pregnancy* to extract relevant data. For states without published Medicaid manuals or with no mention of abortion policies in their manuals, we conducted a secondary search using the state’s Medicaid website search engine, administrative codes and rules, and statutes, applying the same keywords to locate Medicaid abortion policies. We also extracted data from abortion certification forms—official Medicaid documents that must be completed for reimbursement of abortion care—when they were mentioned as a requirement in their respective state policies. We included only documents sourced from official state Medicaid or government websites. All sources referenced were downloaded and archived electronically.

### Data Analysis

We used a thematic analysis approach to identify and categorize the scope of abortion coverage across states.^17^ Two authors (J.W.J. and S.J.H.) performed initial coding on data from 5 randomly selected states to develop preliminary codes. Through group discussion, 4 authors (J.W.J., S.J.H., D.O., and H.K.) refined preliminary codes to create a comprehensive codebook. Subsequently, 2 authors (J.W.J. and S.J.H.) independently coded 5 additional randomly selected states, further refining the codebook. Using the finalized codebook, all data files were double-coded, with discrepancies resolved through documented group discussions. Finally, 4 authors (J.W.J., S.J.H., D.O., and H.K.) independently reviewed all codes to identify key themes and then finalized themes through consensus-based discussion. All coding was completed using Google Sheets spreadsheet software, web version as of July 2024 (Google). All quantitative findings were reported by tallying the number of states in each category.

## Results

### Data Sources

We analyzed a total of 94 documents and websites, with publication dates ranging from March 2005 to February 2024, with most sources updated within the past 5 years (Table). The documents revealed 3 key themes. The main sources of information referenced included state Medicaid manuals (33 states), state administrative rules and codes (11 states), nonmanual Medicaid documents (ie, Medicaid Frequently Asked Questions, Medicaid state plan) (4 states), state statutes (2 states), and an abortion certification form (1 state). Notably, 34 states mandated completion of an abortion certification form or a written statement for Medicaid coverage, with 32 states publishing official forms and 2 states providing sample language (eFigure in Supplement 1). Requirements for form completion varied, typically involving physician certification and sometimes patient signatures and occasionally allowing input from other agency officials such as law enforcement representatives.

**Table.** Characteristics of Sources Referenced for State Medicaid Policies

State^a^	Main source referenced	Date listed	Abortion certification form	Agent filling out form^b^
Alabama	Medicaid manual	Jul 2023	Yes	Physician
Alaska	Medicaid manual	Jan 2019	Yes	Attending physician
Arizona	Medicaid manual	Jan 2024	Not mentioned	NA
Arkansas	Medicaid manual	Feb 2022	Yes	Physician and patient
California	Medicaid manual	Oct 2022	No	NA
Colorado	Medicaid manual	Sep 2023	Yes	Practitioner
Connecticut	Medicaid manual	Oct 2020	Yes	Attending physician
Delaware	Medicaid manual	Jan 2024	Yes	Physician
District of Columbia	Other Medicaid document	May 2018	Not mentioned	NA
Florida	Administrative rules or codes	Jul 2016	Yes	Physician
Georgia	Medicaid manual	Jan 2024	Yes	Physician
Hawaii	Medicaid manual	Jan 2011	Not mentioned	NA
Idaho	Medicaid manual	Feb 2024	Sample documentation provided by state	Physician
Illinois	Medicaid manual	Jun 2021	Not mentioned	NA
Indiana	Medicaid manual	Apr 2023	Not mentioned	NA
Iowa	Medicaid manual	Dec 2021	Yes	Attending provider (in the case of life endangerment or fetal condition)
				Official of law enforcement, public or private health agency, which may include a family physician (in the case of rape or incest)
Kansas	Medicaid manual	May 2023	Yes	Patient and physician
Kentucky	State statutes	Dec 2021	Not mentioned	NA
Louisiana	Medicaid manual	Feb 2012	Not mentioned	NA
Maine	Administrative rules or codes	May 2022	Not mentioned	NA
Maryland	Medicaid manual	Jan 2023	Not mentioned	NA
Massachusetts	Administrative rules or codes	Jun 2022	Yes	Attending practitioner and consulting practitioner (in the case of severe and long-lasting damage to pregnant individual’s physical health)
				Law enforcement or public health agency authority (in the case of rape or incest)
Michigan	Medicaid manual	Jan 2024	Yes	Physician
Minnesota	Medicaid manual	Feb 2024	Yes	Physician
Mississippi	Administrative rules or codes	Oct 2013	Yes	Physician
Missouri	Administrative rules or codes	Feb 2022	Yes	Performing physician
Montana	Medicaid manual	Mar 2021	Yes	Physician
Nebraska	Administrative rules or codes	Jun 2022	Not mentioned	NA
Nevada	Medicaid manual	Jul 2022	Yes	Provider (in the case of life endangerment)
				Recipient, witness 1, witness 2, and physician (in the case of rape or incest)
New Hampshire	Abortion certification form	Jul 2012	Yes	Patient and attending physician
New Jersey	Administrative rules or codes	Feb 2024	Yes	Physician who performed the abortion
New Mexico	Administrative rules or codes	Apr 2022	Not mentioned	NA
New York	Other Medicaid document	Apr 2023	Not mentioned	NA
North Carolina	Medicaid manual	Feb 2024	Yes	Physician
North Dakota	Medicaid manual	Jan 2024	Not mentioned	NA
Ohio	Administrative rules or codes	Mar 2005	Yes	Physician
Oklahoma	Administrative rules or codes	Sep 2021	Yes	Physician performing abortion and patient
Oregon	Other Medicaid document	Jan 2023	Not mentioned	NA
Pennsylvania	Administrative rules or codes	Jun 2023	Yes	Physician
Rhode Island	Medicaid manual	NA	Specific documentation required	Physician
South Carolina	Medicaid manual	Feb 2024	Yes	Physician and patient (in the case of rape or incest)
South Dakota	State statutes	Jul 2022	Not mentioned	NA
Tennessee	Other Medicaid document	Jan 2024	Yes	Physician performing abortion
Texas	Medicaid manual	Sep 2023	Yes	Physician
Utah	Medicaid manual	Jan 2024	Yes	Attending physician performing the procedure and recipient (or legal representative)
Vermont	Medicaid manual	Feb 2019	Yes	Physician
Virginia	Medicaid manual	Oct 2022	Yes	Doctor
Washington	Medicaid manual	Feb 2024	Not mentioned	NA
West Virginia	Medicaid manual	Sep 2022	Yes	Attending physician
Wisconsin	Medicaid manual	Feb 2024	Yes	Physician
Wyoming	Medicaid manual	Jan 2024	Yes	Physician

Abbreviation: NA, not applicable.

^a^ Includes the District of Columbia.

^b^ Direct quotations, as written in each respective state’s abortion certification form or sample documentation.

### Theme 1: Heterogeneity in Scope of Abortion Coverage

Our examination of Medicaid abortion coverage across states reveals substantial heterogeneity in how states define the scope of abortion care coverage. We identified 4 distinct and mutually exclusive categories of clinical coverage, which are summarized in Box 1, theme 1, subthemes 1 to 4. Subtheme 1 comprises policies that closely adhered to the language of the current iteration of the Hyde Amendment. States in this category emphasized coverage for abortions in cases of “a life-endangering physical condition” that would “place the woman in danger of death unless an abortion is performed.” Subtheme 2 includes policies that described life-threatening situations without use of current Hyde wording. State policies in this category used terms such as *save the life*, *life endangerment*, *preservation of life*, and *avert the death* to define the scope of coverage. Subtheme 3 encompasses policies that provided additional abortion care coverage beyond the parameters of the Hyde Amendment, covering all medically necessary abortions or otherwise specified conditions. This category includes policies that provided coverage for abortions deemed medically necessary by the physician, life-threatening psychiatric conditions, fetal anomalies, and other specified clinical conditions not explicitly mentioned in the Hyde Amendment. Subtheme 4 includes policies that provided coverage for all abortions without restrictions.

Box 1. **Illustrative Quotations by State for Scope of Medicaid Abortion Coverage**

**Theme 1: heterogeneity in scope of coverage**

**Subtheme 1: current federal Hyde Amendment wording referenced**

Indiana: “Abortions are covered only if the pregnancy is the result of an act of rape or incest or a case where a woman suffers from a physical disorder, physical injury or physical illness, including a life-endangering physical condition caused by or arising from the pregnancy itself, which would, as certified by a physician, place the individual in danger of death unless an abortion is performed and in compliance with 42 CFR 441.202.”
Nebraska: “Therapeutic abortions are covered only in the case where a woman suffers from a physical disorder, physical injury, or physical illness, including a life-endangering physical condition caused by or arising from the pregnancy itself, that would, as certified by a physician, place the woman in danger of death unless an abortion is performed; therapeutic abortions are also covered in cases of rape or incest.”

**Subtheme 2: life endangerment described without current Hyde Amendment wording**

Arkansas: “Federal regulations prohibit expenditures for abortions except when the life of the mother would be endangered if the fetus were carried to term or for victims of rape or incest....”
Nevada: “Abortion services are covered only for pregnancy resulting from rape or incest or if the procedure is necessary to save the life of the mother.”

**Subtheme 3: coverage for medically necessary conditions and/or conditions specified beyond the Hyde Amendment**

Alaska: “I certify based upon all of the information available to me that the above does not apply, but in my professional medical judgment the abortion procedure was medically necessary to avoid a threat of serious risk to the physical health of the woman from continuation of her pregnancy due to the impairment of a major bodily function including but not limited to one of the following:
  ___ diabetes with acute metabolic derangement or severe end organ damage
  ___ renal disease that requires dialysis treatment
  [Nineteen other specified conditions were omitted for length.]
  ___ physical illness, including a physical condition arising from the pregnancy, or a psychiatric disorder that places the woman in imminent danger of medical impairment of a major bodily function if an abortion is not performed.”
Colorado: “The presence of a psychiatric condition, which represents a serious and substantial threat to the life of the pregnant woman if the pregnancy continues to term….”
Connecticut: “The department shall pay the billing provider for all abortions that a physician certifies as medically necessary whether or not the woman’s life would be endangered by carrying the fetus to term and whether or not the pregnancy is the result of rape or incest. For the purposes of abortion coverage and payment, a physician determines medical necessity.”
Maryland: “Specifically, a physician or surgeon must certify that, based on his or her professional opinion, the procedure is necessary due to one of the following conditions: ...it can be ascertained by the physician with a reasonable degree of medical certainty that the fetus is affected by genetic defect or serious deformity or abnormality….”
New York: “Medicaid also relies on the language from the federal Supreme Court decision Doe V. Bolton to further refine the definition of medically necessary abortions. This decision held that the determination that an abortion is medically necessary ‘is a professional judgment that may be exercised in the light of all factors—physical, emotional, psychological, familial, and the woman’s age—relevant to the well-being of the patient. All these factors may relate to health.’”^18^

**Subtheme 4: all abortions covered**

California: “Abortion is a covered benefit regardless of the gestational age of the fetus. Medical justification and authorization for abortion are not required.”
Maine: “The Department shall provide coverage to MaineCare members for Abortion services that are not covered or reimbursed by Medicaid, including medical services and supplies incidental or preliminary to an Abortion, when performed by a Health Care Professional in a licensed general hospital or outpatient setting.”

**Subtheme 5: rape and incest not mentioned**

South Dakota: “No funds of the State of South Dakota or any agency, county, municipality, or any other political subdivision thereof and no federal funds passing through the state treasury or any agency of the State of South Dakota, county, municipality, or any other political subdivision thereof, shall be authorized or paid to or on behalf of any person or entity for or in connection with any abortion that is not necessary for the preservation of the life of the person upon whom the abortion is performed.”

After categorizing each state into 1 of the 4 categories of clinical coverage as represented by the aforementioned subthemes, we found that there were 18 states in the subtheme 1 category (Hyde Amendment wording), 10 states in the subtheme 2 category (life endangerment described without current Hyde Amendment wording), 17 states in the subtheme 3 category (medically necessary and/or other conditions specified beyond the Hyde Amendment), and 6 states in the subtheme 4 category (all abortions covered) (Figure). All but 2 states explicitly indicated that they either provided coverage for abortions in cases of rape or incest or covered all abortions. Kentucky referenced the Hyde Amendment, thereby implicitly covering abortions in cases of rape or incest but provided no additional details. South Dakota did not mention coverage for rape or incest exceptions (Box 1, theme 1, subtheme 5).

**Figure.**
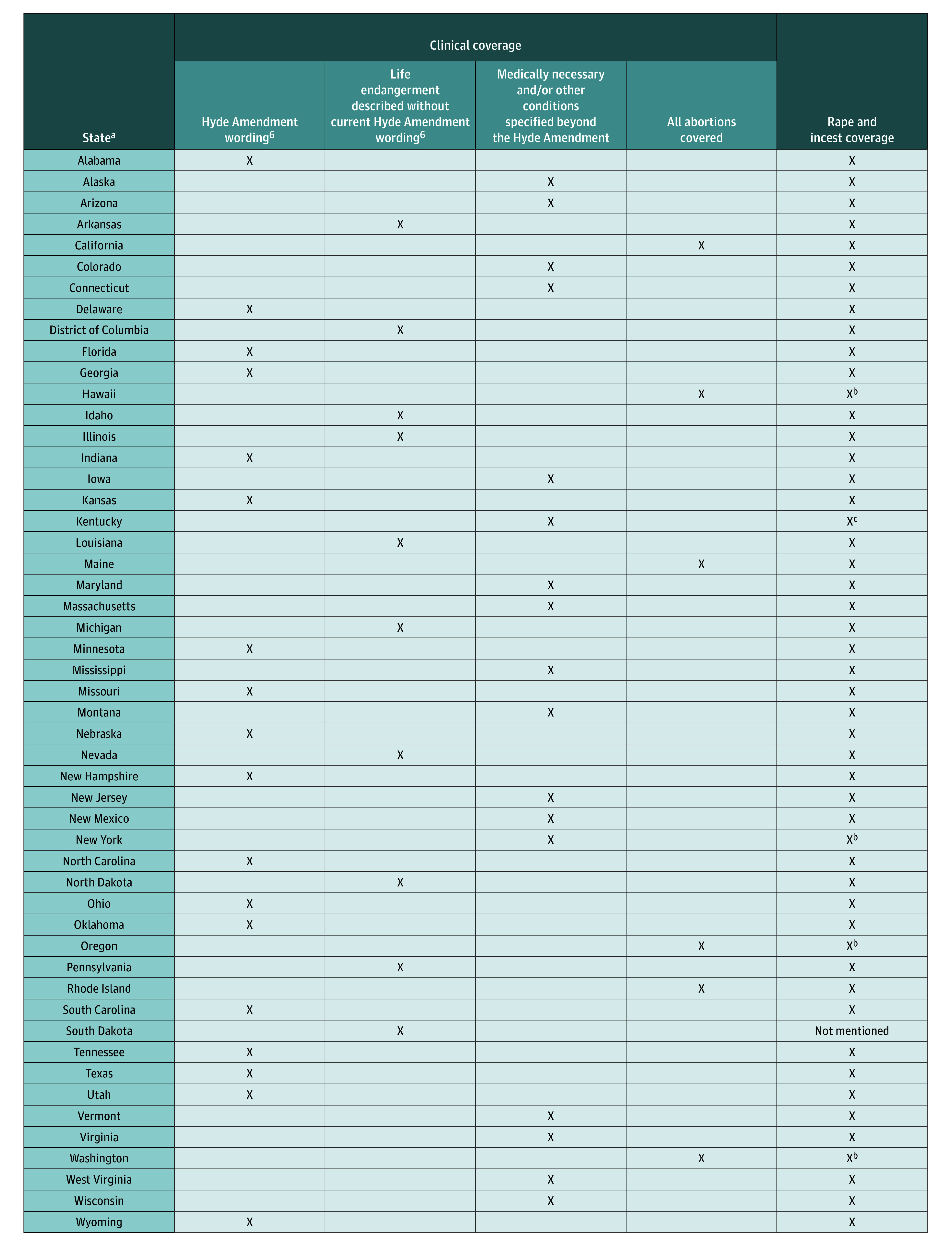
Summary of State Medicaid Abortion Coverage as Written in State Medicaid Policies ^a^Includes the District of Columbia. ^b^Implicitly covered because the state covers all abortions. ^c^Implicitly covered because the state references the Hyde Amendment^6^ without using explicit wording.

States that expanded coverage beyond the parameters outlined in the Hyde Amendment varied widely in their definitions and criteria (Box 1, theme 1, subtheme 3). Some states broadly covered any abortion deemed medically necessary by a physician, regardless of whether the life of the pregnant person was endangered, while others had more defined criteria. The specificity of covered conditions ranged from detailed lists (eg, Alaska provided a list of 22 conditions) to less defined references (eg, in Connecticut, a physician determines whether an abortion is medically necessary). While most states focused on physical health conditions as outlined in the current version of the Hyde Amendment, 3 states (Florida, New Hampshire, and Utah) explicitly excluded nonphysical factors, and 7 states gave consideration to nonphysical factors, such as psychiatric conditions, behavioral health, mental health, and emotional factors. Five states explicitly covered certain fetal anomalies. Three states (New Jersey, New York, and Vermont) specifically cited the language in *Doe v Bolton* of a medically necessary abortion as one that takes into consideration the woman’s age, as well as psychological and familial factors.^18^

### Theme 2: Patient Burdens

While abortion coverage policies vary across states, patients commonly face challenges when attempting to obtain coverage for abortion care (Box 2).

Box 2. **Illustrative Quotations by State for Patient Burdens Found in Medicaid Policies**

**Theme 1: reporting requirements for rape and incest cases**

Arizona: “When the pregnancy is the result of rape or incest, documentation shall be obtained that the incident was reported to the proper authorities, including the name of the agency to which it was reported, the report number (if available), and the date the report was filed. This documentation requirement shall be waived if the treating physician certifies that, in their professional opinion, the member was unable, for physical or psychological reasons, to comply with the requirement....”
Virginia: “Rape or incest cases must be reported to law enforcement, a Virginia Department of Health local health district office in the locality where the patient resides, or the Virginia Department of Health state office.”
Wyoming: “The pregnancy is the result of sexual assault as defined in Wyoming Statute W.S. 6-2-301, which was reported to a law enforcement agency within five (5) days after the assault or within five (5) days after the time the victim was capable of reporting the assault.”

**Theme 2: administrative hurdles**

Arkansas: “[The certification form] must be completed and signed by both the physician and the patient prior to the date of the abortion procedure.”
Indiana: “Medical abortion by oral ingestion of mifepristone and misoprostol requires three separate office visits to complete the procedure. Confirmation of pregnancy status must occur before the Day 1 office visit. The Day 1 office visit must occur after the 18-hour counseling and waiting period required by IC 16-34-2-1.1(a)(1).”
Iowa: “The mother has been given the opportunity to view an ultrasound image of the fetus as part of the standard care before an abortion is performed, and the mother has been provided information regarding the options relative to a pregnancy including continuing the pregnancy to term and retaining parental rights following the child’s birth, continuing the pregnancy to term and placing the child for adoption, and terminating the pregnancy.”

**Theme 3: language and terminology**

Louisiana: “Medicaid only covers an abortion performed by a physician and related hospital charges when it has been determined medically necessary to save the life of the mother or when the pregnancy is the result of rape or incest.”
Mississippi: “‘Serious health risk to the unborn child’s mother’ means that in reasonable medical judgment, she has a condition that so complicates her medical condition that it necessitates the abortion of her pregnancy to avert her death or to avert serious risk of substantial and irreversible physical impairment of a major bodily function, not including psychological or emotional conditions.”
New Hampshire: “Per federal directive, only life endangerment of the mother, not the baby, qualifies.”

#### Reporting Requirements for Rape and Incest Cases

Many states had reporting requirements for abortions due to rape or incest (Box 2, theme 1), with 22 states requiring patients seeking an abortion for this reason to report the incident to authorities (eg, police, courts, and health agencies). Only 14 of these 22 states allowed an option not to report. Six states imposed reporting timelines, ranging from 3 to 60 days. West Virginia required reporting within 48 hours of the abortion.

#### Administrative Hurdles

Many states imposed additional administrative requirements (Box 2, theme 2). Examples of such requirements include mandating that abortion certification forms are signed prior to abortion care being provided, requiring multiple office visits for medication abortions, and imposing mandatory ultrasound viewing and counseling on pregnancy continuation and adoption.

#### Language and Terminology

States often used fetus-centered language in their policies (Box 2, theme 3), with some states using terms like *unborn child* or *baby*. There was also frequent use of the term *mother*, especially in phrases like *death of the mother* or *life of the mother*.

### Theme 3: Physician Responsibilities

In addition to patient burdens, physicians must fulfill an extensive range of responsibilities for Medicaid abortion coverage (Box 3).

Box 3. **Illustrative Quotations by State for Physician Burdens Found in Medicaid Policies**

**Theme 1: onus placed on physician to determine eligibility**

Delaware: “One of those requirements is that, in order for Medicaid to reimburse for an abortion, a physician must certify that a woman suffers from a physical disorder, physical injury, or physical illness, including a life-endangering physical condition caused or arising from the pregnancy itself that would place the woman in danger of death unless an abortion is performed.”
Kentucky: “In the attending physician’s reasonable medical judgment, the abortion was necessary to prevent the death of the pregnant woman or to avoid a serious risk of the substantial and irreversible impairment of a major bodily function of the pregnant woman.”
North Carolina: “Medicaid requires the physician performing the abortion to submit certification (refer to Subsection 5.3) in writing attesting to the fact that in his/her professional judgment the beneficiary was a victim of rape.”
Pennsylvania: “Where a physician has certified in writing and documented in the patient’s record that the life of the woman would be endangered if the pregnancy were allowed to progress to term. The decision as to whether the woman’s life is endangered is a medical judgment to be made by the woman’s physician.”

**Theme 2: documentation and administrative requirements**

Alabama: “Providers must use these fillable consent forms with the digital submission of Consent Forms and supporting documentation. Any form received that is not in a fillable format will be returned to the provider.”
Alaska: “Providers must submit a signed Certificate to Request Funds for Abortion form when abortion services are performed for Medicaid and [Denali KidCare] recipients. An original signature of the attending physician is required; a facsimile or photocopied signature or signature of the physician’s authorized representative will not be accepted.”
Colorado: “An evaluation by a licensed physician specializing in psychiatry must accompany the claim for reimbursement for the abortion if a psychiatric condition represents a serious and substantial threat to the pregnant woman’s life if the pregnancy continues to term.”
Iowa: “The reason for the abortion must be identified on form 470-0836, Certification Regarding Abortion. This form must be attached to the claim for payment, along with the following documentation:

- The operative report
- The pathology report
- Laboratory reports
- The ultrasound report
- The physician’s progress notes
- Other documents that support the diagnosis identified on the claim.”

Minnesota: “‘Medical necessity’ means (1) the signed written statement of two physicians indicating the abortion is medically necessary to prevent the death of the mother....”
Utah: “In addition to the above conditions, Medicaid reimbursement for abortion services is allowed only when:

- Prior authorization is obtained….”

#### Onus Placed on Physician to Determine Eligibility

Many states gave physicians the responsibility of determining eligibility for abortion care coverage (Box 3, theme 1). Thirty-eight states explicitly required physician certification and justification for clinical conditions warranting coverage. Of the 32 states requiring abortion certification forms, many asked physicians to explain the reason for the abortion. Requirements varied by state, with some states such as Delaware mandating physician certification of life-endangering conditions, while others such as Wisconsin required certification of sexual assault or incest.

#### Documentation and Administrative Requirements

Physicians also had to fulfill various documentation and administrative requirements to ensure coverage (Box 3, theme 2), such as completing certification forms, providing medical records, and coordinating with other parties such as additional physicians or law enforcement representatives. Many policies provided specific submission instructions, such as using designated portals and requiring original signatures. Several state policies also explicitly mandated prior authorization for abortion coverage.

## Discussion

In this qualitative study of state Medicaid abortion policies, we demonstrated wide variability in the scope of Medicaid abortion coverage across states, with coverage ranging from strict Hyde Amendment adherence to comprehensive abortion coverage. State differences in policies extend beyond clinical criteria to include variation in reporting requirements, documentation standards, and administrative procedures, which may further complicate access to Medicaid abortion coverage. Notably, this research represents the first, to our knowledge, comprehensive examination of the specific language used in state abortion policies, primarily derived from official Medicaid documents.

The heterogeneity in policy language that we found can be, in part, attributed to the numerous versions of the Hyde Amendment that have been enacted since 1979. Our analysis found remnants of past versions such as the phrase *save the life of the mother*, rape or incest reporting requirements, or requirements of certification by 2 physicians in current state policies, creating a patchwork of outdated language that does not always align with current federal guidelines.^19,20,21^ Furthermore, we discovered that policies were often scattered across multiple documents or inconsistent within documents from the same state. These inconsistencies may, in part, stem from a lag between updated federal policies and their incorporation at the state level, suggesting a lack of centralized oversight of state Medicaid policies.

Our policy analysis also revealed a pattern of barriers and requirements for coverage that poses substantial challenges for patients. For cases of rape and incest, patients faced mandated reporting to authorities in 22 states, with some states imposing narrow time frames. These requirements pose substantial challenges, as disclosing sexual assault or abuse to authorities can be difficult and traumatizing due to fear of retaliation, mistrust in authorities, societal stigma, fear of not being believed, and feelings of shame or guilt, among other factors.^22,23,24^ While the HHS instructed states to waive reporting requirements if patients were deemed not able to comply with them,^7,8^ not all states explicitly included this option in their policies.

Patients faced additional barriers stemming from state policies imposed on patients regardless of type of insurance, including mandatory ultrasound viewings, counseling on continuing the pregnancy, and imposed waiting periods before abortion procedures. Research has shown that these friction strategies do not increase decision certainty but instead delay care, increase financial strains, and elevate distress for patients seeking abortion services.^25,26,27^ Furthermore, the use of terms such as *baby* and *unborn child* in select state policies is medically inaccurate and inherently biased,^28,29^ which may impact both patients’ and physicians’ perspectives and choices. Similarly, policies that refer to the pregnant person as the mother can contribute to abortion stigma as well and potentially serve as a deterrent for people seeking abortion care.^30,31^

Physicians face extensive administrative responsibilities related to abortion care for Medicaid beneficiaries, including completing abortion certification forms, providing extensive medical documentation, coordinating with third parties for specific records, and filing prior authorizations. While certification requirements share similarities to those for other services requiring authorization, the combination of vaguely and inconsistently worded abortion policies and the increasingly politicized and litigious environment surrounding abortions may put abortion care physicians in a uniquely challenging position. Establishing life endangerment and medical necessity in the context of abortions is also often difficult and nuanced.^32^ Abortion care physicians have reported that the lack of clarity in abortion policies and fear of legal repercussions have led to a reluctance to sign abortion certification forms as well as delays and limitations in abortion care that would otherwise be medically necessary.^32,33,34,35^ Such delays may create practical barriers to access in states with time-sensitive abortions due to restrictions on gestational age or for patients needing an abortion for a life-threatening condition. Filing required prior authorizations is also not always feasible in the setting of emergent abortion care.

Other administrative tasks that were uncovered included obtaining original signatures, uploading documents to specific portals, and adhering to formatting specifications. In general, abortion care physicians report that administrative demands consume an excess amount of staff time and resources and lead to bureaucratic inefficiencies.^35^ Such a process, in conjunction with bureaucratic inefficiencies, has rendered past physician efforts to obtain Medicaid abortion coverage futile, with some physicians reporting that more than half of qualifying abortions in their practice go unreimbursed.^35^ Interventions to streamline forms and protocols were reported as beneficial for the reimbursement process.^35^

Importantly, Medicaid abortion coverage policies disproportionately affect vulnerable populations, particularly racial and ethnic minority groups and individuals with disabilities, who are overrepresented among both Medicaid recipients and those seeking an abortion.^36,37,38^ As a result, states with more restrictive Medicaid abortion coverage as well as onerous patient and physician requirements likely have a disproportionate impact on these marginalized populations, potentially exacerbating existing health inequities.

Our findings should be understood within the context of the broader policy landscape. Access to abortion through Medicaid is limited not only by program-specific rules but also by legal developments—particularly the total or near-total abortion bans enacted by many states since the *Dobbs* decision. The heterogeneity of Medicaid abortion policies and myriad requirements reported in this study may represent another layer of complexity and confusion for patients and physicians. Although enacting and implementing improvements may be challenging given that Medicaid programs operate under complex federal–state partnerships with competing priorities, there are opportunities for interventions that could enhance coverage clarity and reduce administrative burden.

One improvement could be the creation of a centralized tool that allows users to look up the most up-to-date state Medicaid scope of abortion coverage and requirements. As laws and policies continue to change, a tool like this could help patients and physicians remain aware of the most current regulations and requirements. Ideally, policy-makers would also develop and implement uniform guidelines to increase clarity of Medicaid abortion coverage requirements. One example of this would be to ensure reporting requirements of rape and incest can be waived in extenuating circumstances, as previously outlined by the HHS,^7,8^ or to provide guidance to eliminate it altogether. We caution, however, that any changes to language defining the scope of coverage could instead be used as an opportunity to stray from statutorily mandated coverage and impose unlawful limitations through additional conditions on coverage or opaque procedural hurdles. Finally, we recommend that states streamline Medicaid abortion reimbursement processes, such as simplifying forms, reducing required steps, and providing clear instructions for navigating the reimbursement process, to lessen administrative burden for both physicians and state Medicaid agencies. These measures that focus on transparency, clarity, and efficiency are first steps toward minimizing complexity and confusion surrounding Medicaid abortion coverage for both patients and abortion care providers—steps that may ultimately improve access to essential reproductive health care services for marginalized populations.

### Limitations

This study has some limitations. Data were most recently extracted and updated in February 2024, but given the ever-changing nature of Medicaid policies and implications of the *Dobbs* decision, we acknowledge that updates may have occurred since then. We also recognize that Medicaid abortion access is constrained by greater systems at play, including federal and state allocations and evolving abortion laws that exist beyond the Medicaid program itself. Furthermore, despite implementing a systematic data-extraction process and a consistency check between 2 data extractors, there is a possibility that some policies were overlooked. Our study also relied on publicly available Medicaid documents and may not represent state Medicaid policies that are not publicly available. Finally, while our analysis identifies substantial policy language variations creating implementation challenges, we acknowledge that the findings primarily document policy heterogeneity rather than directly measuring clinical outcomes.

## Conclusions

The findings of this qualitative study of state Medicaid abortion policies demonstrate a heterogeneous scope of abortion coverage among states and substantial barriers for both patients and physicians in accessing Medicaid coverage for abortion care. Measures that improve transparency, clarity, and efficiency may help patients and physicians navigate complex coverage policies and improve access to essential abortion care for vulnerable populations.

## Notes

Supplement 1. **eFigure.** Protocol for Collecting Documents on State Medicaid Abortion Policies

Supplement 2.
Data Sharing Statement

## References

[1] Jones RK, Kirstein M, Philbin J. Abortion incidence and service availability in the United States, 2020. Perspect Sex Reprod Health. 2022;54(4):128-141. doi:10.1363/psrh.12215 36404279 PMC10099841

[2] *Dobbs v Jackson Women’s Health Organization*, 597 US 215 (2022).

[3] *Roe v Wade*, 410 US 113 (1973).

[4] Curhan T. State bans on abortion throughout pregnancy. Ephross P, ed. Guttmacher Institute. July 7, 2025. Accessed July 30, 2025. https://www.guttmacher.org/state-policy/explore/state-policies-abortion-bans

[5] Pub L No. 94-439, 90 Stat 1434, Sec 209 (1976). Accessed July 29, 2025. https://www.congress.gov/94/statute/STATUTE-90/STATUTE-90-Pg1418.pdf

[6] Pub L No. 118-47, 138 Stat 703, Sec 507 (2024). Accessed July 29, 2025. https://www.congress.gov/118/plaws/publ47/PLAW-118publ47.pdf

[7] Richardson SK. Letter to State Medicaid Director. Health Care Financing Administration, Department of Health & Human Services. December 28, 1993. Accessed July 30, 2025. https://healthlaw.org/wp-content/uploads/2022/10/DSMD-12-28-1993-re-Hyde.pdf

[8] Richardson SK. Letter to State Medicaid Director. Center for Medicaid and State Operations, Department of Health & Human Services. February 12, 1998. Accessed July 30, 2025. https://www.medicaid.gov/federal-policy-guidance/downloads/smd021298.pdf

[9] Jarlenski M, Hutcheon JA, Bodnar LM, Simhan HN. State Medicaid coverage of medically necessary abortions and severe maternal morbidity and maternal mortality. Obstet Gynecol. 2017;129(5):786-794. doi:10.1097/AOG.0000000000001982 28383380 PMC5400718

[10] Vilda D, Wallace ME, Daniel C, Evans MG, Stoecker C, Theall KP. State abortion policies and maternal death in the United States, 2015–2018. Am J Public Health. 2021;111(9):1696-1704. doi:10.2105/AJPH.2021.306396 34410825 PMC8589072

[11] Heil SKR, Caglayan K, Castillo G, . The impact of state Medicaid coverage of abortion on people accessing care in three states. Perspect Sex Reprod Health. 2024;56(3):255-268. doi:10.1111/psrh.12275 39074851 PMC11605993

[12] Jones RK. Medicaid’s role in alleviating some of the financial burden of abortion: findings from the 2021-2022 Abortion Patient Survey. Perspect Sex Reprod Health. 2024;56(3):244-254. doi:10.1111/psrh.12250 38366736 PMC11605995

[13] McDonnell J, Jarlenski M, Borrero S, Vinekar K. Association of availability of state Medicaid coverage for abortion with abortion access in the United States. Obstet Gynecol. 2022;140(4):623-630. doi:10.1097/AOG.0000000000004933 36075060

[14] Carrión F, Duffy C, Mendoza C. Abortion coverage under Medicaid. National Health Law Program; April 27, 2022. Accessed July 4, 2024. https://healthlaw.org/wp-content/uploads/2022/04/FINAL-Abortion-Coverage-Under-Medicaid.pdf

[15] Dennis A, Blanchard K. Abortion providers’ experiences with Medicaid abortion coverage policies: a qualitative multistate study. Health Serv Res. 2013;48(1):236-252. doi:10.1111/j.1475-6773.2012.01443.x 22742741 PMC3589964

[16] Khidir H, Topping C, Dalton VK, Lindau ST, Venkatesh AK. Mystery shopper study of state Medicaid coverage for out-of-state abortion care. JAMA Netw Open. 2023;6(11):e2343569-e2343569. doi:10.1001/jamanetworkopen.2023.43569 37966843 PMC10652153

[17] Kiger ME, Varpio L. Thematic analysis of qualitative data: AMEE Guide No. 131. Med Teach. 2020;42(8):846-854. doi:10.1080/0142159X.2020.1755030 32356468

[18] *Doe v Bolton*, 410 US 179 (1973).

[19] Pub L No. 103-112, 107 Stat 1113, Sec 509 (1993). Accessed July 30, 2025. https://www.congress.gov/103/statute/STATUTE-107/STATUTE-107-Pg1082.pdf

[20] Pub L No. 95-480, 92 Stat 1586, Sec 210 (1978). Accessed July 30, 2025. https://uscode.house.gov/statutes/pl/95/480.pdf

[21] Liu EC, Shen WW. The Hyde Amendment: an overview. Congressional Research Service; July 20, 2022. Accessed February 19, 2025. https://www.congress.gov/crs_external_products/IF/PDF/IF12167/IF12167.2.pdf

[22] Sable MR, Danis F, Mauzy DL, Gallagher SK. Barriers to reporting sexual assault for women and men: perspectives of college students. J Am Coll Health. 2006;55(3):157-162. doi:10.3200/JACH.55.3.157-162 17175901

[23] Seibold-Simpson SM, McKinnon AM, Mattson RE, . Person- and incident-level predictors of blame, disclosure, and reporting to authorities in rape scenarios. J Interpers Violence. 2021;36(9-10):NP4788-NP4814. doi:10.1177/0886260518795171 30139298

[24] Wolitzky-Taylor KB, Resnick HS, McCauley JL, Amstadter AB, Kilpatrick DG, Ruggiero KJ. Is reporting of rape on the rise? a comparison of women with reported versus unreported rape experiences in the National Women’s Study—Replication. J Interpers Violence. 2011;26(4):807-832. doi:10.1177/0886260510365869 20522886

[25] Jovel I, Cartwright AF, Ralph L, Upadhyay UD. Abortion waiting periods and decision certainty among people searching online for abortion care. Obstet Gynecol. 2021;137(4):597-605. doi:10.1097/AOG.0000000000004313 33706354 PMC7984762

[26] Karasek D, Roberts SC, Weitz TA. Abortion patients’ experience and perceptions of waiting periods: survey evidence before Arizona’s two-visit 24-hour mandatory waiting period law. Womens Health Issues. 2016;26(1):60-66. doi:10.1016/j.whi.2015.10.004 26626710

[27] Morse JE, Charm S, Bryant A, Ramesh S, Krashin J, Stuart GS. The impact of a 72-hour waiting period on women’s access to abortion care at a hospital-based clinic in North Carolina. N C Med J. 2018;79(4):205-209. doi:10.18043/ncm.79.4.205 29991607

[28] ACOG Guide to Language and Abortion. The American College of Obstetricians and Gynecologists. Accessed July 4, 2025. https://www.acog.org/contact/media-center/abortion-language-guide

[29] Mikołajczak M, Bilewicz M. Foetus or child? abortion discourse and attributions of humanness. Br J Soc Psychol. 2015;54(3):500-518. doi:10.1111/bjso.12096 25418861

[30] Kumar A, Hessini L, Mitchell EM. Conceptualising abortion stigma. Cult Health Sex. 2009;11(6):625-639. doi:10.1080/13691050902842741 19437175

[31] Norris A, Bessett D, Steinberg JR, Kavanaugh ML, De Zordo S, Becker D. Abortion stigma: a reconceptualization of constituents, causes, and consequences. Womens Health Issues. 2011;21(3)(suppl):S49-S54. doi:10.1016/j.whi.2011.02.010 21530840

[32] Sabbath EL, McKetchnie SM, Arora KS, Buchbinder M. US obstetrician-gynecologists’ perceived impacts of post-Dobbs v Jackson state abortion bans. JAMA Netw Open. 2024;7(1):e2352109-e2352109. doi:10.1001/jamanetworkopen.2023.52109 38231510 PMC10794934

[33] Lilly AG, Newman IP, Bjork-James S. Our hands are tied: abortion bans and hesitant medicine. Soc Sci Med. 2024;350:116912. doi:10.1016/j.socscimed.2024.116912 38723584

[34] Suran M. Treating cancer in pregnant patients after Roe v Wade overturned. JAMA. 2022;328(17):1674-1676. doi:10.1001/jama.2022.13668 36173620

[35] Kacanek D, Dennis A, Miller K, Blanchard K. Medicaid funding for abortion: providers’ experiences with cases involving rape, incest and life endangerment. Perspect Sex Reprod Health. 2010;42(2):79-86. doi:10.1363/4207910 20618746

[36] Race and ethnicity of the national Medicaid and CHIP population in 2020. Centers for Medicare & Medicaid Services; 2020. Accessed July 3, 2024. https://www.medicaid.gov/medicaid/data-and-systems/downloads/macbis/2020-race-etncity-data-brf.pdf

[37] Drake P, Burns A. Medicaid. Working-age adults with disabilities living in the community. KFF. January 4, 2024. Accessed November 5, 2024. https://www.kff.org/medicaid/issue-brief/working-age-adults-with-disabilities-living-in-the-community/

[38] Kortsmit K, Nguyen AT, Mandel MG, . Abortion surveillance—United States, 2021. MMWR Surveill Summ. 2023;72(9):1-29. doi:10.15585/mmwr.ss7209a1 37992038 PMC10684357

