# Supplementary material for: State-Level Variation in and Barriers to Medicaid Abortion Coverage
Source: JAMA Netw Open. 2025 Sep 8;8(9):e2530804. doi: 10.1001/jamanetworkopen.2025.30804 (PMC12418122; doi:10.1001/jamanetworkopen.2025.30804)
Supplement: Supplement 1. — eFigure. Protocol for Collecting Documents on State Medicaid Abortion Policies [file jamanetwopen-e2530804-s001.pdf]

Supplemental Online Content

Jiang JW, Ho SJ, Suttiratana SC, et al. State-level variation in and barriers to Medicaid abortion coverage. *JAMA Netw Open*. 2025;8(9):e2530804.
doi:10.1001/jamanetworkopen.2025.30804

eFigure. Protocol for Collecting Documents on State Medicaid Abortion Policies

This supplemental material has been provided by the authors to give readers additional information about their work.

© 2025 Jiang JW et al. *JAMA Network Open*.

eFigure. Protocol for Collecting Documents on State Medicaid Abortion Policies

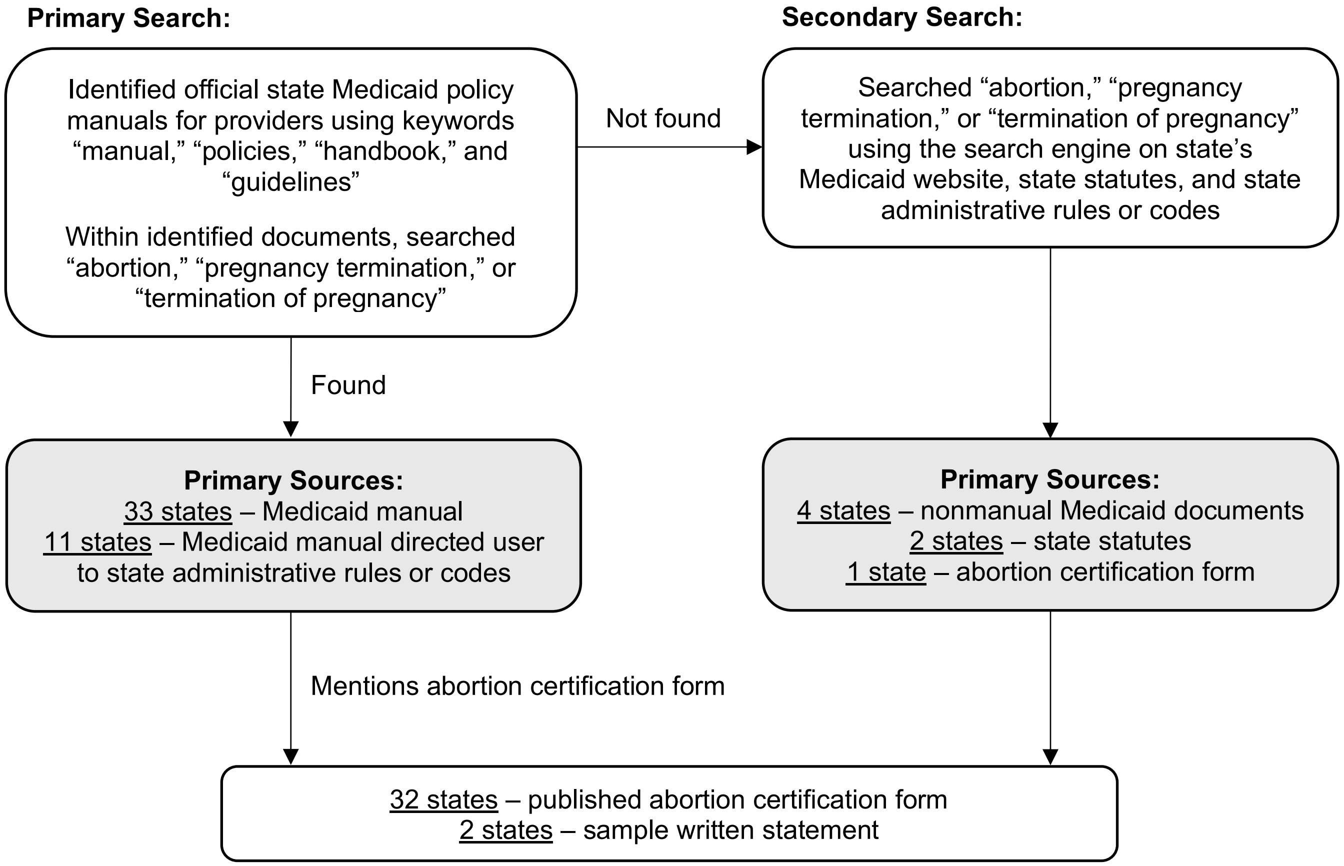

© 2025 Jiang JW et al. *JAMA Network Open*.
