# Supplementary material for: State-Level Variation in and Barriers to Medicaid Abortion Coverage
Source: JAMA Netw Open. 2025 Sep 8;8(9):e2530804. doi: 10.1001/jamanetworkopen.2025.30804 (PMC12418122; doi:10.1001/jamanetworkopen.2025.30804)
Supplement: Supplement 2. — Data Sharing Statement [file jamanetwopen-e2530804-s002.pdf]

Data Sharing Statement

Jiang. State-Level Variation in and Barriers to Medicaid Abortion Coverage. *JAMA Netw Open*. Published September 08, 2025. doi:10.1001/jamanetworkopen.2025.30804

Data
Data available: Yes
Data types: Data (not involving human participants)
How to access data: Email address: jasmine.jiang@yale.edu
When available: With publication

Supporting Documents
Document types: None

Additional Information
Who can access the data: Researchers whose proposed use of the data has been approved.
Types of analyses: For approved, related research.
Mechanisms of data availability: With investigator support, after approval of a proposal, and signed data access agreement.
